# National Yellow Fever Relief Commission

**Published:** 1878-11-20

**Authors:** Jno. M. Woodworth

**Affiliations:** Chairman of Committee to Edit Report; Washington, D. C.


### [illegible]IONAL YELLOW-FEVER RELIEF COMMISSION.

EXECUTIVE COMMITTEE:

| | |
|---|---|
| A. R. Shepherd, Chm'n | John T. Mitchell. |
| Wm. Dickson, Sec'y. | A. S. Solomons. |
| L. J. Davis, Treas'r. | Simon Wolf. |
| Arthur MacArthur. | B. H. Warner. |
| George Hill, Jr. | John F. Cook. |
| Leonard Whitney. | J. M. Woodworth, M. D. |

WASHINGTON, D. C., Nov. 1, 1878.

*To the Yellow-Fever Relief Associations, Com., Etc.:*

At a meeting of the Executive Committee of the National Yellow-Fever Relief Commission, held on October 30, 1878, the following resolutions were adopted, and directed to be forwarded to the various Relief Associations throughout the United States:—[The interesting Preamble we omit for want of space.—ED.]

"*Resolved,* That all relief committees and other organizations which have contributed in aid of the yellow-fever sufferers of the South during the present epidemic, be requested to communicate to this commission the gross amonnt they have collected, and in what manner they have disposed of their funds, together with such other information as will give a history of their benevolent work.

"*Resolved,* That a committee of three, of which Surgeon-General Woodworth, of the Marine Hospital service, shall be chairman, be designated by hechair to prepare and edit this report, and that they be empowered to employ such clerical assistance as may be deemed necessary.

In accordance with the foregoing resolutions, Relief Associations, Committees, etc., are requested to fill the appended blank, detach and return it at as early a date as possible to Dr. John M. Woodworth, P. O. Box 33, Washington, D. C. In addition to this, such items of information as you may have from newspaper clippings, letters of correspondents, or extracts from letters, which will be of use in making up the full history of this relief work, are respectfully solicited.

Signed JNO. M. WOODWORTH,
Chairman of Committee to Edit Report.

